# Epidemiologic and clinical characteristics of human bocavirus infection in children hospitalized for acute respiratory tract infection in Qingdao, China

**DOI:** 10.3389/fmicb.2022.935688

**Published:** 2022-08-10

**Authors:** Wenjing Wang, Renzheng Guan, Ziran Liu, Feng Zhang, Rui Sun, Sitong Liu, Xiaoyan Shi, Zhilei Su, Rongxiang Liang, Kangyu Hao, Zhaoguo Wang, Xianming Liu

**Affiliations:** ^1^Department of Epidemiology and Health Statistics, The College of Public Health of Qingdao University, Qingdao, China; ^2^Department of Pediatrics, Affiliated Hospital of Qingdao University, Qingdao, China; ^3^Qingdao Municipal Center for Disease Control and Prevention, Qingdao, China; ^4^Department of Neurosurgery, Qingdao Municipal Hospital, Qingdao, China

**Keywords:** human bocavirus, acute respiratory infections, viral infection, bacteria, *Stenotrophomonas maltophilia*, multiplex real-time PCR

## Abstract

Persistent infection and prolonged shedding of human bocavirus 1 (HBoV1) in children have been reported, and the role of HBoV1 as a sole causative pathogen in acute respiratory infection (ARI) is yet to be established. While the reported prevalence of HBoV infection varies due to different detection methods and sampling criteria, determining the viral and bacterial etiology of HBoV infection using multiplex real-time PCR is yet to be reported. Herein, we aimed to further explore the pathogenicity of HBoV in patients with ARI by screening the viral and bacterial infections in children with ARI in Qingdao and comparing the epidemiological, clinical characteristics, and etiological results. Human bocavirus was identified in 28.1% of the samples, and further sequencing analysis of the detected HBoV confirmed 96.4% as HBoV1. The rate of HBoV as a single viral infection was 75%, and the rate of coinfection with bacteria was 66.1%, suggesting the need for continued monitoring of HBoV in children with ARIs. Clinical characterization suggested that HBoV infection may affect the function of organs, such as the liver, kidney, and heart, and the blood acid–base balance. Additionally, it is essential to promote awareness about the importance of disinfection and sterilization of the hospital environment and standardizing operations. The interactions between HBoV and other pathogens remain to be investigated in further detail in the future.

## Introduction

Acute respiratory infections (ARIs) are among the most common illnesses among hospitalized children worldwide. The burden of ARIs on health care facilities is remarkable. ARIs rank fourth among the world's leading causes of death (Murray et al., 2020). There have been several major outbreaks and even pandemics caused by infectious respiratory viruses over the past two decades, namely, the severe acute respiratory syndrome coronavirus (SARS-COV), Middle East respiratory syndrome coronavirus (MERS-COV), new H1N1 pandemic influenza (H1N1), severe acute respiratory syndrome coronavirus 2 (SARS-CoV-2), and other respiratory viruses, which have caused great harm (Dat et al., 2021) and posed considerable public health threats to human society (Zar and Ferkol, 2014). ARIs are a complex and heterogeneous group of diseases, which are spread by bacteria, fungi, and viruses. Respiratory syncytial virus (RSV), Influenza virus (IFV), human parainfluenza virus (HPIV), human adenovirus (HAdV), human metapneumovirus (HMPV), human rhinovirus (HRV), human coronaviruses (HCoV), and other respiratory viruses (Venter et al., 2011; Lukšić et al., 2013; Feng et al., 2014; Xu et al., 2018) are common pathogens of ARIs, according to the current research findings. Bacteria can be primary or secondary causative agents of ARIs, where secondary bacterial infections often occurred after the initial viral infection has reduced or suppressed the immune response of the respiratory tract. Common bacteria that can cause ARIs include *Streptococcus pneumoniae* and *Haemophilus influenzae* (Albrich et al., 2004; Murphy et al., 2009).

Human bocavirus (HBoV) is an icosahedral, linear, single-stranded DNA virus with a size between 18 and 26 nm. It was originally discovered by Allander et al. in (2005) in respiratory tract specimens (Ingrand, 2006). The full length of the HBoV genome is 4.7–5.7 kb (Cotmore and Tattersall, 2014). Because the genome has a bilateral hairpin structure, the full length of its genome cannot be accurately determined. There are three open-reading frames (ORF1, ORF2, and ORF3), wherein ORF1 and ORF2 encode nonstructural proteins NS1 and NP1, and ORF3 encodes capsid proteins VP1 and VP2. Generally, it is believed that NS1 and NP1 are relatively conserved in HBoV. NS1 and NP1 are important for viral DNA replication (Huang et al., 2012; Shen et al., 2015); these regions are typically utilized as target regions for the detection of HBoV (Zeng et al., 2011). Comparatively, VP1 and VP2 have greater mutational diversity and are therefore commonly used for phylogenetic analysis of HBoV (Blinkova et al., 2009). Owing to the current NS1-based taxonomy, the four human bocaviruses in the genus *Bocaparvovirus* belong to two different species. HBoV1 and HBoV3 belong to *Primate bocaparvovirus* 1, while HBoV2 and HBoV4 belong to *Primate bocaparvovirus* 2 (Soderlund-Venermo, 2019). HBoV3 is a recombinant between HBoV1 and HBoV4, where the VP1 gene of HBoV3 is more similar to HBoV4 (Kapoor et al., 2010). HBoV1 has been primarily found in samples from the respiratory tract; however, three other variants (HBoV 2–4) have been identified in samples from the gastrointestinal tract and seem to be associated with gastroenteritis (Guido et al., 2016). There is uncertainty regarding the involvement of HBoV2–4 in respiratory infections, although they have been detected in samples from the respiratory tract, albeit at much lower levels. The transmission of HBoV is most likely to occur *via* the fecal-oral and respiratory routes (Kumthip et al., 2021). HBoV is widespread worldwide and endemic throughout the year; the infection tends to peak in winter and spring (Blinkova et al., 2009; Kumar et al., 2011). In addition, owing to the long persistence of HBoV DNA in the nasopharynx for weeks and months, even up to a year (Martin et al., 2015) after initial infection (Ma et al., 2012), an accurate diagnosis of acute infection of HBoV1 usually needs additional serological evidence. HBoV1 is often detected in conjunction with other pathogens; the frequency of coinfections is high, and the clinical symptoms are more severe than those associated with monoinfections (Sun et al., 2019). However, there have been instances of life-threatening infections caused by a single infection (Ziemele et al., 2019). Furthermore, it is not yet known whether HBoV is a true ARI pathogen, making the diagnosis of acute HBoV1 infection very challenging.

In the current study, the aim is to evaluate the prevalence, epidemiologic, and clinical characteristics of HBoV among children with ARIs in Qingdao.

## Materials and methods

### Patients and sample collection

We studied children under the age of 12 years with ARIs who were hospitalized between October and December 2018 at the Pediatric Respiratory and Cardiovascular Department of a hospital in Qingdao. ARIs were defined as having (1) at least one of the following conditions: fever, abnormal white blood cell (WBC) differentials, leukocytosis, or leukopenia; and (2) at least one of the following symptoms/signs: cough, chills, expectoration, nasal congestion, sore throat, chest pain, tachypnea, or abnormal pulmonary breath sounds. Patients with confirmed diagnoses of noninfectious respiratory diseases were excluded. All the selected patients have been diagnosed with ARI.

### Specimen collection

On the day of admission (before treatment), nasopharyngeal swabs were collected, placed in a viral transfer medium, and immediately transferred to the molecular microbiology laboratory of the hospital, where seven respiratory viruses were investigated using direct immunofluorescence assay (DFA respiratory virus screening and ID kit, Diagnostic Hybrids, USA). The samples remaining after the antigen tests were stored at −80°C before being transferred to the Qingdao Municipal Center for Disease Control and Prevention for a one-step multiplex real-time RT-PCR screening of respiratory virus pathogens. Sample information included collection date, direct immunofluorescence assay (DFA) test date, names of nurses who collected the samples, names of lab staff who performed the DFA, and names of doctors who prescribed the DFA. The following variables were exported from digital clinical records: age, sex, demographics, date of admission, diagnosis, clinical symptoms, physical examination, and clinical diagnosis, together with detailed laboratory and imaging examination results.

### Respiratory viruses and bacteria detection

Total nucleic acids were extracted from 200 μl nasopharyngeal swabs using the MagNA Pure 96 DNA and Viral NA Small Volume Kit (Roche, Basel, Switzerland) according to the manufacturer's instructions. Subsequently, we used the one-step multiplex real-time RT-PCR kit (Neuro-Hemin Biotech Co, Ltd, China) to detect and screen the presence of 16 respiratory viruses (Li et al., 2021a), namely, human parainfluenza virus 1 (HPIV-1), human parainfluenza virus 2 (HPIV-2), human parainfluenza virus 3 (HPIV-3), human parainfluenza virus 4 (HPIV-4), influenza A virus (IFV-A), influenza B virus (IFV-B), human adenovirus (HAdV), respiratory syncytial virus A/B (RSV A/B), human rhinovirus A/B/C (HRV A/B/C), human enterovirus (HEV), human bocavirus 1/2/3/4 (HBoV 1/2/3/4), human metapneumovirus (HMPV), human coronavirus OC43 (HCoV OC43), human coronavirus 229E/NL63 (HCoV-229E/NL63), and the novel Middle East respiratory syndrome coronavirus (MERS).

Specimens positive and negative for HBoV were further tested for 16 bacterial pathogens (Li et al., 2021b) using a one-step multiplex fluorescence real-time PCR kit (Neuro-Hemin Biotech Co, Ltd, China). The types of bacteria tested were *Mycoplasma pneumoniae, Klebsiella pneumoniae, Legionella pneumophila, Streptococcus pneumoniae, Staphylococcus aureus, Chlamydia pneumonia, Pseudomonas aeruginosa, Moraxella catarrhalis, Bordetella pertussis, Haemophilus influenzae, Acinetobacter baumannii, Mycobacterium tuberculosis/Mycobacterium avium, Serratia marcescens, Bordetella parapertussis*, and *Stenotrophomonas maltophilia*.

### Sanger sequencing

The HBoV-positive samples were selected, and the TaKaRa Ex Taq DNA Polymerase kit (Code: RR001A) was used for panbocavirus nested PCR amplification of the 576-bp HBoV VP1/2 gene. The first-round PCR reaction conditions were as follows: 95°C for 2 min, followed by 10 cycles, 95°C for 35 s (58°C for 1 min, minus 0.5°C for each cycle), 72°C for 1 min, followed by 30 cycles, 95°C for 30 s, 54°C for 45 s, and 72°C for 45 s, and finally extended for 10 min at 72°C. Similar conditions were repeated except that the initial annealing temperatures in the first and second groups of PCR cycles in the second round were changed to 60 and 58°C, respectively. The primer sequences used are shown in Table 1 (Kapoor et al., 2010; Lu, 2013). The amplified products were then sent to sequencing companies for sequencing.

**Table 1:** Primers used in this study.

**Virus**	**Primer name**	**Sequence (5^′-3′^)**
HBoV	HBoV-1F	CGCCGTGGCTCCTGCTCT
	HBoV-1R	TGTTCGCCATCACAAAAGATGTG
	HBoV-2F	GGCTCCTGCTCTAGGAAATAAAGAG
	HBoV-2R	CCTGCTGTTAGGTCGTTGTTGTATGT

### Phylogenetic analysis

The recent prevalent representative strains of HBoV1 worldwide were obtained from GenBank and used together with the sequences obtained in this study to construct a comparative phylogenetic tree. MAFFT was used to perform the alignments (Katoh and Standley, 2013). Phylogenetic trees were generated using the Molecular Evolutionary Genetics Analysis (MEGA) software v X and the maximum likelihood method under the most appropriate model of its substitution determined with the jModel test v2.1.4 (Darriba et al., 2012). Bootstrap probabilities for 1,000 iterations were calculated to evaluate confidence estimates.

The consensus sequences of HBoV1 strains obtained in this study were deposited in GenBank under accession numbers ON263364–ON263392 and ON994387–ON994398.

### Statistical analysis

All analyses were conducted using SPSS Statistics version 20.0 (IBM SPSS Inc., Chicago, USA). The measured data that conform to a normal distribution are expressed in terms of the mean ($\bar{X}$), and the data are compared using a *t*-test. Measurements that fall within a skewed distribution are expressed as medians (IQR), and the data are compared using a rank-sum test. A categorical variable is expressed in numbers or percentages, and proportions are compared using Pearson's chi-square or Fisher's exact tests. Two-sided *P-*values < 0.05 were considered significant.

### Ethics statement

The studies involving human participants were reviewed and approved by the Regional Ethics Committee of the Qingdao Centers for Disease Control and Prevention Institutional Review Boards. Written informed consent to participate in this study was provided by the participant's legal guardian/next of kin.

## Results

### Study patients

In the period between October and December 2018, 263 children were hospitalized, of which 64 cases (24.3%) were excluded due to missing respiratory specimens, incomplete case data, clinical diagnosis not ARI, and insufficient sample volume to extract nucleic acid. Therefore, 199 cases (75.7%) were selected for analysis in this study. Their median age was 3 years [interquartile range (IQR) = 1–6 years]. There were 114 male and 85 female children, with a gender ratio of 1.34:1.32. It was determined that 32.7% of patients were aged <2 years, 37.2% were aged 2–5 years, and 30.1% were aged ≥5 years. The demographic information and basic clinical features of the cases are listed in Table 2.

**Table 2:** Demographic information and basic clinical features of the cases.

**Characteristics**	***N* (%)**
**Gender**
Male	114 (57.3)
Female	85 (42.7)
**Age**
<2 years old	65 (32.7)
2–5 years old	74 (37.2)
≥5 years old	60 (30.1)
**Symptoms**
Fever	77 (38.7)
Cough	197 (99.0)
Vomiting or diarrhea	13 (6.5)
Wheezing	57 (28.6)
Rales	136 (68.3)

### Detection of pathogens

In terms of virus detection proportion, the most commonly encountered viral pathogen was HBoV (28.1% of cases, 56/199), followed by RSV (37, 18.6%), HRV (5, 2.5%), HAdV (4, 2.0 %), HPIV-4 (4, 2.0%), IFV-A (3, 1.5%), HEV (1, 0.5%), HCoV-229E (1, 0.5%), HPIV-1 (1, 0.5%), and HPIV-2 (1, 0.5%).

In light of the proportion of positive detections of bacteria, *S. pneumoniae* was the most frequently detected bacterium, accounting for 35.7% (71/199) of total patients, followed by *M. catarrhalis* (54, 27.1%), *H. influenzae* (37, 18.6%), *S. maltophilia* (30, 15.1%), *S. aureus* (23, 11.6%), *A. baumannii* (20, 10.1%), *M. tuberculosis*/*M. avium* (18, 9.0%), *M. pneumoniae* (17, 8.5%), *K. pneumoniae* (12, 6.0%), *B. pertussis* (11, 5.5%), *C. pneumoniae* (7, 3.5%), *P. aeruginosa* (3, 1.5%), *L. pneumophila* (1, 0.5%), *S. marcescens* (1, 0.5%), and *B. parapertussis* (1, 0.5%).

**Figure 1:**
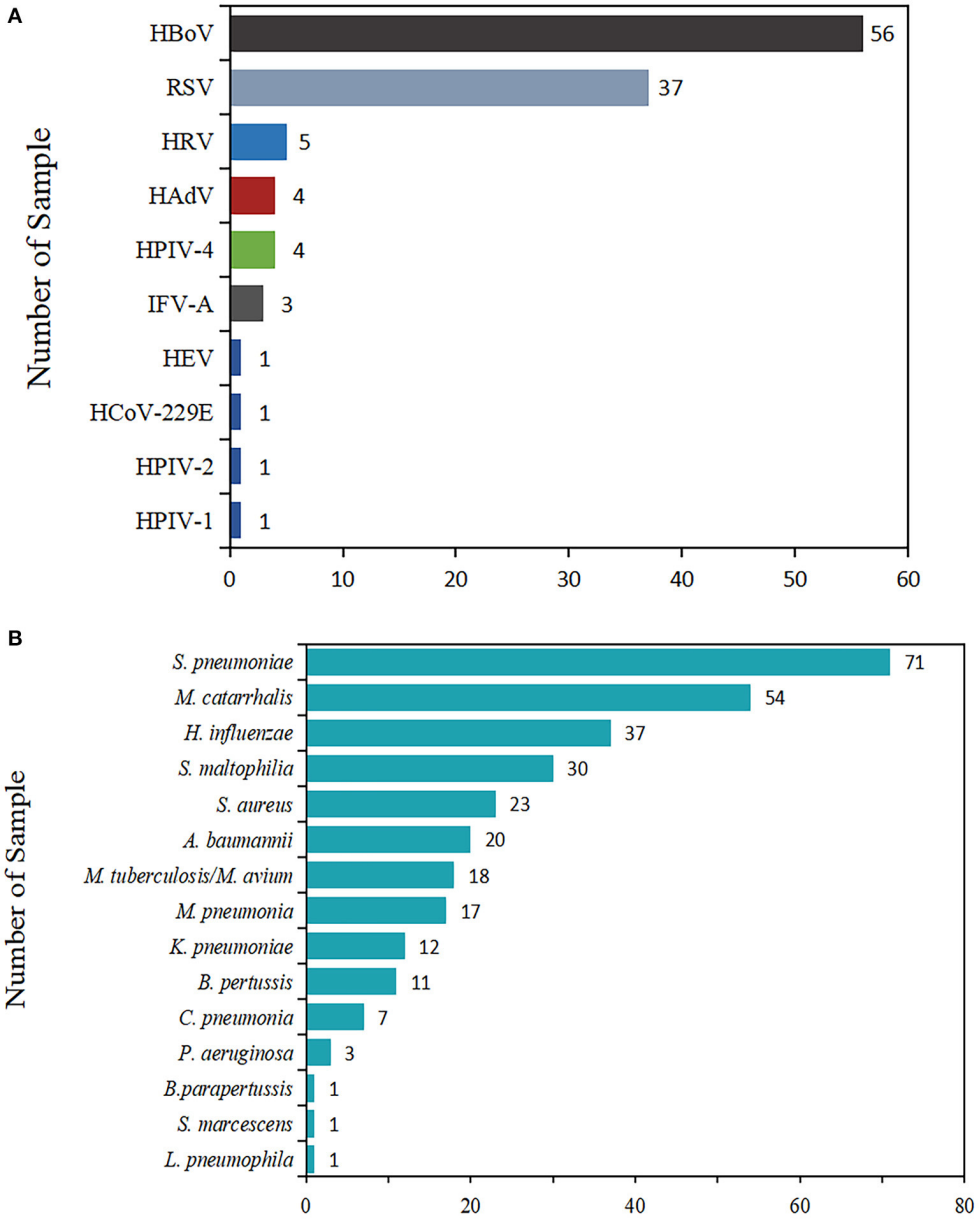
Total number of viruses and bacteria detected in children with ARIs. The length of the color bar and the number marked behind are the number of pathogens detected. **(A)** The detection of viruses. **(B)** The detection of bacteria.

The positive rates, pathogen spectrum, and clinical characteristics were compared and are presented (Figure 1).

### Pathogen detection and clinical characteristics of patients with or without detection of HBoV

A total of 199 individuals were selected for the study, 56 of whom were HBoV-positive. The remaining 143 individuals did not test positive for HBoV. The study subjects were categorized into two groups depending on whether they tested positive for HBoV. In the HBoV-positive group, 42 cases of HBoV single viral infection were reported with an infection rate of 75%. The number of HBoV-positive patients coinfected with other viruses was 14, with an infection rate of 25%. Of the 14 viral coinfected patients, 11 were coinfected with only one virus, and 3 were coinfected with two viruses. RSV was the most common respiratory virus to coinfect with HBoV (*n* = 9), followed by HRV (*n* = 3). Table 3 provides information on other viruses that were detected. RSV detection ranked second (37, 18.6%) only to HBoV in the study population; its detection rate was 16.1 and 19.6% in the HBoV-positive and HBoV-negative groups, respectively. The detection rate of HRV differed slightly between the two groups, being second (5.4%) to RSV in the HBoV-positive group and being third together with IFV-A (1.4%) after RSV, HPIV-4, and HAdV (2.1%) in the HBoV-negative group.

**Table 3:** The type of HBoV coinfected with other viruses.

	**Type of co-infection**	***n* (%)**
Two types of viruses	HBoV + RSV	6 (10.7)
(*n* = 11, 19.6%)
	HBoV + HRV	1 (1.8)
	HBoV + HPIV1	1 (1.8)
	HBoV + HPIV2	1 (1.8)
	HBoV + HPIV4	1 (1.8)
	HBoV + HAdV	1 (1.8)
Three types of viruses (*n* = 3, 5.4%)	HBoV + RSV + HRV	2 (3.6)
	HBoV + RSV + IFV-A	1 (1.8)

In the HBoV-negative group, 34 (23.8%) of the patients had other viral infections, and 109 samples (76.2%) were negative for viral infection. There were 30 cases (21.0%) in the HBoV-negative group without bacterial infection and 113 cases (79.0%) with a concomitant bacterial infection. The bacterial test detected 39 cases with bacterial infection (69.6%) among the 56 patients in the HBoV-positive group and 17 cases without bacterial infection (30.4%). Among the 39 children with bacterial infections, bacterial monoinfection was present in 14 cases, bacterial dual infections were present in 18 cases, bacterial triple infections were present in six cases, and four bacterial species were detected in 1 case. The combination of bacteria detected was more dispersed, with the most detected being a mixed infection of *S. pneumoniae* and *M. catarrhalis* with a detection rate of 8.9% (5/56), followed by a single infection of *H. influenzae* and mixed infection of *S. pneumoniae and S. maltophilia*, both with a detection rate of 5.4% (3/56). The remaining bacterial infections are shown in Supplementary Table 1. In addition, *S. pneumoniae* had the highest detection rate in both groups (32.1 and 37.1%). Following this was *M. catarrhalis, H. influenzae*, and *S. maltophilia*, with detection rates of 23.2 and 28.7, 16.1 and 19.6, and 12.5 and 16.1% in the two groups, respectively.

**Figure 2:**
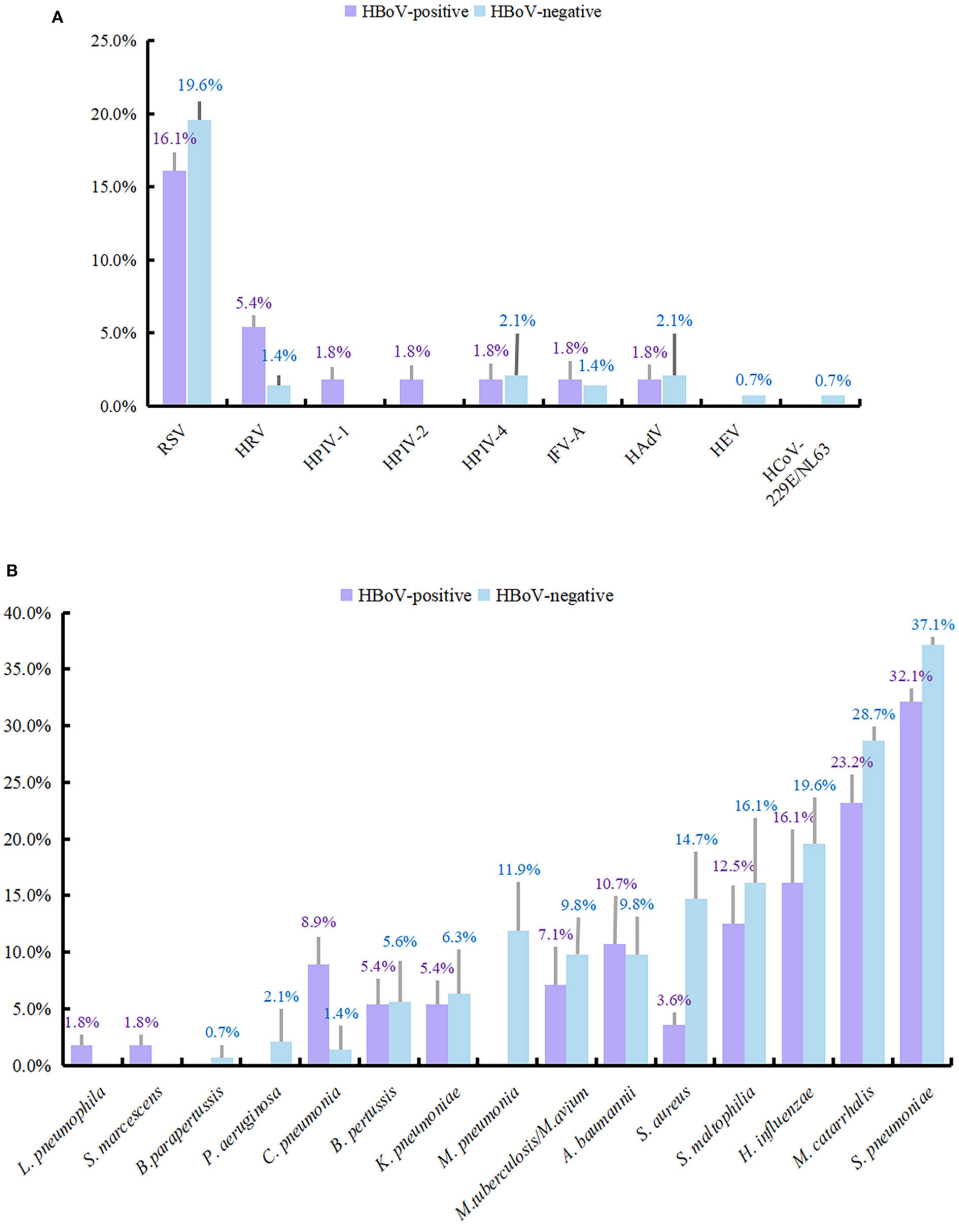
Detection rate of viruses and bacteria in HBoV-infected group and uninfected group. The length of the color bar and the following number indicate the detection rate of each pathogen, with its positive number as the numerator and the number of people in each group as the denominator. **(A)** The detection rate of viruses. **(B)** The detection rate of bacteria.

The detection rates of other viruses and bacteria are shown in Figure 2.

Among the 56 patients with and the 143 patients without the detection of HBoV, 29 (51.8%) and 85 (59.4%) were males and 27 (48.2%) and 58 (40.6%) were females, respectively, with no significant differences in gender between the two groups of patients (χ^2^ = 0.964, *P* = 0.326). It was found that children ≥5 years (37.5%) had a significantly higher infection rate for HBoV, followed by children under 2 years (33.9%) and children 2–5 years old (28.6%; χ^2^ = 2.989, *P* = 0.224).

The most prevalent symptom in HBoV-positive and HBoV-negative patients was cough, with rales coming second in both groups. Based on the statistical analysis, the concentrations of abnormal lactate dehydrogenase (LDH), urea (URE), creatine kinase isoenzyme (CK-MB), and Mg in the plasma of HBoV-positive patients were different from those in the plasma of HBoV-negative patients, and the difference was significant. The elevated level of aspartate aminotransaminase (AST) was significant in HBoV-positive patients with the median value close to the upper range in this liver function index.

Furthermore, the number of HBoV-negative patients with increased lung markings was significantly higher than that of HBoV-positive patients. When other laboratory measures, such as the count of white blood cells (WBC) and red blood cells (RBC) in routine blood tests, and the concentration of Na in electrolyte indexes, were compared, however, there was no significant difference between HBoV-positive and HBoV-negative patients (Table 4).

**Table 4:** Clinical characteristics of ARI patients with or without HBoV.

**Variable**	**HBoV-positive (*n* = 56)**	**HBoV-negative (*n* = 143)**	**χ^2^/*Z*/*t***	***P*-value**
**Gender**
Male	29 (51.79%)	85 (59.44%)	0.964	0.326
Female	27 (48.21%)	58 (40.56%)		
**Age**
<2 years old	19 (33.93%)	46 (32.17%)	2.989	0.224
2–5 years old	16 (28.57%)	58 (40.56%)		
≥5 years old	21 (37.50%)	39 (27.27%)		
**Clinical features**
Fever	23 (41.07%)	54 (37.76%)	0.186	0.666
Cough	55 (98.21%)	142 (99.30%)	0.477	0.490
Wheezing	12 (21.43%)	45 (31.47%)	1.985	0.159
Rales	39 (69.64%)	97 (67.83%)	0.061	0.805
Vomiting or diarrhea	5 (8.93%)	8 (5.59%)	0.733	0.392
WBC, 10^9^/L	7.84 (5.83, 10.91)	7.26 (5.41, 9.55)	1.562	0.118
AST, U/L	31.40 (22.38, 38.28)	26.00 (22.00, 31.60)	2.146	0.032^*^
ALT, U/L	13.80 (10.75, 21.98)	13.30 (10.60, 17.90)	0.753	0.452
CRP, mg/L	3.02 (0.52, 17.10)	1.48 (0.50, 15.80)	0.888	0.374
LDH, U/L	279.50 (249.00, 326.75)	261.00 (233.00, 307.00)	2.193	0.028^*^
ADA, U/L	20.00 (18.00, 23.00)	20.00 (15.00, 23.00)	0.832	0.405
EOS, 10^9^/L	0.12 (0.05, 0.21)	0.11 (0.02, 0.24)	0.249	0.803
RBC, 10^9^/L	4.45 ± 0.46	4.50 ± 0.41	0.716	0.475
URE, mmol	2.52 (1.91, 3.03)	2.79 (2.10, 3.45)	2.298	0.022^*^
URIC, umol/L	227.00 (172.75, 254.50)	197.00 (165.00, 250.00)	1.407	0.159
CK-MB, U/L	25.60 (20.00, 36.10)	22.70 (17.60, 30.00)	1.956	0.050^*^
Cys-C, mg/L	0.59 (0.49, 0.76)	0.62 (0.50, 0.79)	0.097	0.923
HCO_3_, mmol/L	21.35 (18.35, 22.78)	21.30 (19.70, 22.70)	1.027	0.305
Na, mmol/L	139.20 ± 2.03	139.04 ± 1.72	0.571	0.569
Mg, mmol/L	1.04 (0.96, 1.13)	1.0 1 (0.93, 1.09)	2.049	0.040^*^
Hospitalization days	7.00 (6.00, 9.00)	7.00 (5.00, 8.00)	2.108	0.035^*^
Increased lung markings	30 (53.57%)	109 (76.22%)	9.805	0.002^*^
Patchy opacities	32 (57.14%)	63 (44.06%)	2.762	0.097
Linear opacities	5 (8.93%)	4 (2.80%)	2.227	0.136
Air bronchogram sign	5 (8.93%)	2 (1.40%)	4.687	0.030^*^

* P-values ≤ 0.05 were considered to be statistical significant.

Fever: T ≥ 37.5°C (axillary temperature).

Reference levels: WBC(4~10), AST(15~40), ALT(9~50), CRP(0~5), LDH(120~250), ADA(4~18), EOS(0.02~0.52), RBC(3.75~5.5), URE(3.1~8.0), URIC(89.2~416), CK-MB(0~17), Cys-C(0.6~1.55), HCO_3_(23~31), Na(137~147), and Mg(0.75~1.02.

### Comparison of the clinical characteristics of HBoV-positive patients between single infection and coinfections with high and low HBoV Ct-values

The median HBoV Ct-value in 56 HBoV-positive patients was 32 (IQR 30–34), and 13 patients (23.2%) in the HBoV-positive group had no other viruses and bacteria detected in their samples. Among eight single HBoV infection patients with Ct-values ranging from 32 to 35, and five single HBoV infection patients with Ct-values ranging from 24 to 30, all were confirmed to be HBoV1. We further divided the HBoV-positive patients into four groups for analysis: high Ct-values with single HBoV infection (*n* = 8), high Ct-values with coinfections (*n* = 29), low Ct-values with single HBoV infection (*n* = 5), and low Ct-values with coinfections (*n* = 14). The levels of Mg were significantly different, while the concentrations were within or slightly above the reference level of this biochemical index. In the imaging results, the highest portion was found in linear opacities (3/8, 37.50%) of high Ct-values in the single-infection group, and in increased lung markings (20/29, 68.97%) of high Ct-values in the coinfection group (Supplementary Table 2).

### Clinical characteristics of HBoV-positive patients infected alone or in combination with other viruses

Based on the comparison of demographic information and clinical characteristics of HBoV-positive patients with single infection and mixed infection, there was a significant difference in gender distribution (*P* = 0.045), but no difference in the age distribution (*P* = 0.686). A notable feature of the clinical presentation was the occurrence of vomiting or diarrhea in five children in the group infected with only HBoV and none in the group infected with a combination of other viruses. The mixed-infected population exhibited a higher proportion of wheezing (42.86%) than the single-infected population (14.29%). The statistical analysis of laboratory examination indicators revealed significant differences in abnormal aspartate aminotransaminase (AST), adenosine deaminase (ADA), eosinophil (EOS), RBC, cystatin-C (Cys-C), and HCO_3_ levels between the two groups (*P* < 0.05). The indexes in the single-infection group were generally higher than those in the mixed-infection group, including EOS, RBC, and HCO_3_; however, the median levels of the indexes in the two groups were within normal limits, except for HCO_3_. The mixed-infection group showed higher levels of ADA and Cys-C than the single-infection group. The median ADA levels were above the normal range, while the median Cys-C levels were within the normal range. In the imaging results, the proportion of confirmed pneumonia in the single-infection group (59.52%) was higher than that in the mixed-infection group (21.43%), and the difference was significant (*P* = 0.014), while there was no difference in the distribution of other basic imaging features.

A comparison of other clinical indicators is shown in Supplementary Table 3.

### Clinical characteristics of HBoV-positive patients with or without bacterial infection

A list of the demographic and clinical characteristics of HBoV patients with a concurrent mixed bacterial infection (Supplementary Table 4) is given in the following section. We found no significant differences in gender (*P* = 0.201) or age groups (*P* = 0.297) between patients with a bacterial infection and patients without a bacterial infection. There was no significant difference (*P* > 0.05) in clinical presentations, such as fever, cough, and wheezing between patients infected with bacteria and those who were not infected. Furthermore, there were no significant differences in WBC, AST, RBC, CK-MB, and other clinical measurements between the two groups. A comparison of imaging characteristics in the two groups did not reveal any significant difference.

### Clinical characteristics of HBoV-positive patients with or without *S. maltophilia*

The 56 patients who were HBoV-positive were divided into two groups: those infected with *S. maltophilia* (*n* = 7) and those not infected with *S. maltophilia* (*n* = 49). In terms of gender and age, there was no significant difference between the two groups (*P* > 0.05). An interesting finding in the study was that patients in the uninfected *S. maltophilia* group had a significantly greater number of rales in their lungs than those in the infected *S. maltophilia* group (*P* < 0.05). The difference in blood Na content between the two groups was also significant (*P* < 0.05). However, there was no significant difference between the two groups regarding laboratory indicators, such as RBC, LDH, and Mg, and imaging elements, such as increased lung markings and air bronchogram sign (*P* > 0.05; Supplementary Table 5).

### Phylogenetic analysis

**Figure 3:**
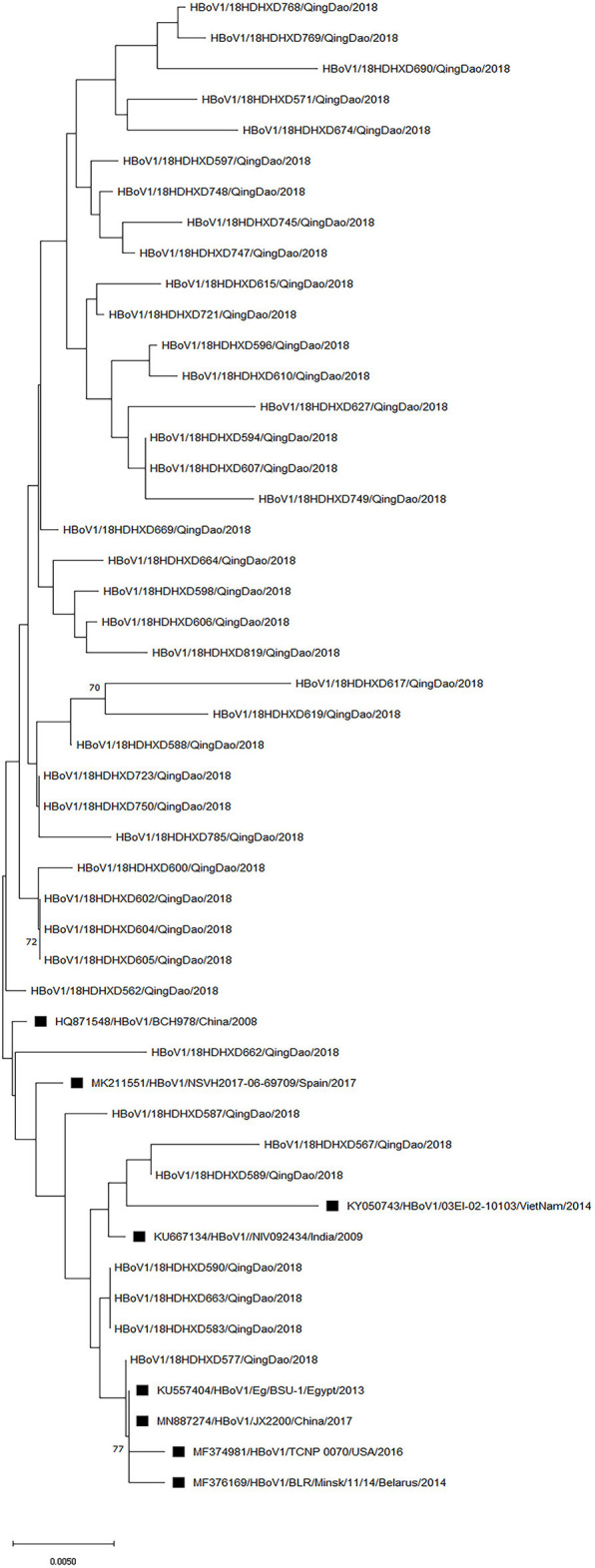
Phylogenetic analysis of the partial VP1/VP2 nucleotide sequences of 41 HBoV1 strains from patients. A phylogenetic tree with 1,000 bootstrap replicates was generated using the maximum likelihood method with MEGA X software. Only bootstrap values >70% are shown on the branch. HBoV1 sequences from Genbank are marked with ■.

Since the VP1/VP2 region of the HBoV genome has the largest variation, particularly at its 3′ end (Kesebir et al., 2006), we chose to target this part of the region for amplification. We were able to amplify and sequence 54 out of 56 HBoV-positive samples, and genotyping based on the BLAST (basic local alignment search tool) could identify all these 54 strains as HBoV1. Among these 54 samples, the sequencing results of some samples were shortened and could not be analyzed together with the other samples, so the phylogenetic analysis was performed on 41 representatives out of the 54 sequences obtained in this study (Figure 3). HBoV1 strains from India, Spain, Belarus, China, USA, Egypt, and Vietnam collected from the respiratory tract and stool samples were selected from the GenBank database (https://www.ncbi.nlm.nih.gov/genbank/) for comparative phylogenetic analysis. The newly detected strains in this study shared 96.95–100% nucleotide sequence identity and 95.11–100% amino acid sequence identity with the above reference strains. The phylogenetic analysis showed that the strains in this study were genetically close to each other, with nucleotide identity (96.88–100%) and amino acid identity (94.99–100%). One strain in this study showed 100% identity in partial VP1/VP2 region at the nucleotide level with the HQ871548 strain, which was from the nasopharyngeal aspirate sample collected in Beijing of northern China after the 2008 Olympic Games.

## Discussion

Modern society is increasingly being globalized, providing fertile grounds for the emergence and spread of infectious diseases. Similar to the outbreak of COVID-19 in Wuhan, China, in 2019, this epidemic is raging around the globe today, burdening the human race tremendously (Hu et al., 2021). There is an urgent need to focus on the detection and research of pathogens causing respiratory tract infections. Identifying pathogens with accuracy, precision, and sensitivity has become of paramount importance. In recent years, one-step multiplex real-time RT-PCR, one of the molecular biological methods, has achieved widespread recognition for its use in pathogen detection. The efficiency of the one-step multiplex real-time RT-PCR is greater than that of the conventional RT-PCR, because it is quicker, more sensitive, and can detect more targets at one time, which saves samples, labor, and cost (Zhang et al., 2020).

In this study, we used the one-step multiplex real-time RT-PCR and multiplex real-time PCR to screen for pneumonia-related viruses and bacteria in 199 children hospitalized for ARIs from October to December 2018 in a hospital in Qingdao. There was a detection rate of 45.2% of respiratory viruses among them, which is slightly greater than the detection rate of respiratory viruses (34.8%) in the ARIs children surveillance project conducted in 106 Chinese cities by Li et al. (2021c), which may be comparable in terms of the sample size and distribution of the study population.

The overall detection rate of HBoV was 28.1%, which was significantly higher than that reported by Ji et al. (2021) (10.0%) who tested lower respiratory tract samples of 878 children in Ningxia and also significantly higher than that of Saudi Arabia (7%) (Abdel-Moneim et al., 2018), Kuwait (1.9%) (Madi and Al-Adwani, 2020), and the United Kingdom (8.66%) (Bagasi et al., 2020). This is slightly lower than the detection rate of HBoV (51.7%) reported by Petrarca et al. (2020) who tested samples of 60 children with ARI in the Rome area. In recent years, it has been suggested that bronchoalveolar lavage fluid has the advantage of high detection accuracy and low contamination rate compared with pharyngeal swab samples in the analysis of infection with pneumonia pathogens (Liu et al., 2019; Yang et al., 2020). This may explain the results of this study, since our respiratory samples were mainly nasopharyngeal swabs. In addition, although the use of the one-step multiplex real-time RT-PCR and two-step RT-PCR did not substantially affect the detection results of HBoV (DNA virus), the sensitivity of the real-time fluorescent multiplex PCR was higher than that of the conventional multiplex PCR. Moreover, our study only enrolled patients during the winter season; using annual data on children with ARI in Qingdao will include more patients, which might provide more comprehensive data comparable to those of current published studies. *S. pneumoniae* was the predominant pathogen detected in the study population. The infection rate reached 35.7%, which is higher than the detection rate of 13.33% for *S. pneumoniae* in infants presenting with ARIs in Ningbo reported by Zhang et al. (2021) and the detection rate of 29.9% for *S. pneumoniae* in the Chinese ARIs Child Surveillance Study for the period 2009–2019 (Li et al., 2021c). The detection rate of *S. pneumonia* has always been at the forefront of ARIs in children around the world, regardless of the differences in detection rates (Simusika et al., 2015; Wei et al., 2015; Li et al., 2021c).

Notably, there were no severe cases reported in the HBoV-positive patients in this study, and no mechanical air ventilation or ICU admission was recorded. The median hospitalization was 7 days. The differences observed between the pathogen etiology and clinical characteristics of HBoV-positive and HBoV-negative patients are presented below. HBoV, RSV, and HRV were detected in high frequencies in both groups, which is in agreement with previous studies (Madi and Al-Adwani, 2020; Ji et al., 2021; Nantachit et al., 2021). As indicated in the results, HBoV infection was more common in children ≥ 5 years old as opposed to most studies at this stage where HBoV1 is more prevalent in children under 2 years of age. This suggests that children ≥5 years old should be considered a priority study group, since this group has relatively higher immunity than children under 2 years old, which might help identify more risk factors for HBoV1 infection in different immune populations. In patients with HBoV infection, respiratory symptoms, such as cough, rales, or wheezing, remain the most common symptoms, which are consistent with previous reports (Ji et al., 2021; Zhang et al., 2021). In HBoV-positive patients, AST, LDH, URE, CK-MB, and Mg levels were significantly different from those in HBoV-negative patients. According to the results of previous studies, AST, URE, and CK-MB were the biochemical indexes of liver dysfunction (Pilut et al., 2022), kidney injury (Zammel et al., 2022), and heart injury (Pilut et al., 2022), respectively. Elevated LDH levels were also often found in patients with myocarditis (Kostik et al., 2022). It cannot be ruled out that HBoV may affect these organs simultaneously, but it is equally possible that these observations are attributable to indirect immune responses of various organs to pathogens (Izumikawa, 2016). Though the median AST level was within the reference range and no patient was diagnosed with acute hepatitis in the study, a periodical follow-up monitoring of liver indexes, including bilirubin, in future research would certainly be beneficial to investigate any potential chronic liver damage in ARI patients infected with HBoV in Qingdao.

In this study, the mixed infection rate of HBoV with other viruses was 25%, which is much lower than the mixed viral infection rate of HBoV in most studies of nasopharyngeal swab samples at this stage, including Moreno et al. (2016) (54.5%) in Panama, Lekana-Douki et al. (2018) (84.4%) in Gabon, and Calvo et al. (2016) (75%) in Spain. Notably, the single viral infection rate in this study is much higher than that in similar studies (Abdel-Moneim et al., 2018; Abozahra et al., 2020; Madi and Al-Adwani, 2020; Ji et al., 2021). In the absence of serological confirmation and lack of evidence of viremia or HBoV mRNA in peripheral blood cells, we still cannot rule out the possibility that the high detection rate of HBoV monoinfection was caused by a recent HBoV infection or persistent HBoV1 viral shedding in the upper respiratory tract of the Qingdao hospitalized children enrolled in this study. Further epidemiological analysis of the temporal and spatial distribution and potential contact links of patients, together with variation in strain sequences, excluded the possibility that the high positive rate of HBoV1 was a witness to HBoV1 outbreaks in Qingdao. One aspect regarding the Ct-value analysis is that the current study used the remaining nasopharyngeal swab samples after the DFA tests; standardized fresh nasopharyngeal aspirate samples might provide an accurate interpretation of the viral load using Ct-values. Even with the above limitation, the positive rate of HBoV in the study patients was 28.1%, with 96.4% being confirmed to be HBoV1 using sequencing; thus, suggesting the prevalence of HBoV1 in healthy and sick children in Qingdao needs to be investigated. According to previous studies, there is no significant difference in the clinical characteristics of HBoV1 infection alone or as a mixed infection (Petrarca et al., 2020; Ji et al., 2021). Both HCO_3_ and ADA were out of the normal range in the study patients, and ADA is one of the important judgment indexes of tuberculous pleurisy (Fielli et al., 2021), although the ADA levels in this study were only slightly higher than the normal reference range and far less than the standard for the determination of tuberculous pleurisy. Some of the patients in this study were infected with *M. tuberculosis/M. avium*, and therefore, the clinical significance of ADA in the infected HBoV population remains to be determined. However, timely implementation of ADA level monitoring in ARIs patients infected with HBoV can achieve early prevention, diagnosis, and treatment of tuberculous pleurisy, and ensure subsequent control and prevention at the epidemiological level. The difference in URE levels between the two groups was significant, and the median of the group infected with HBoV alone was slightly lower than the lower limit of the reference range, which indicates the possibility of sampling error limited by the small sample size. In contrast, the proportion of imaging results confirmed that pneumonia in the single-infection group was higher than that in the mixed-infection group, and there was a significant difference between the two groups. In addition, low Ct-values of HBoV-positive patients had a lower rate of lung markings observed than high Ct-values of HBoV-positive patients (31.58 vs. 64.86%, *P* = 0.0018), suggesting that a single HBoV infection may not aggravate lung disease in nonsevere patients (He et al., 2021; Ogimi et al., 2021).

The number of mixed infections of HBoV with bacteria is not widely studied. However, in this study, bacteria were detected in 69.6% of HBoV-positive children with upper respiratory infections, and the bacterial infection rate was also higher than that observed in previous studies (Cia et al., 2021; Ji et al., 2021). We tried to explain this phenomenon in terms of the difference in detection methods and the ability to achieve screening for more target pathogens with the assays selected for this study. While previous studies chose more bacterial culture methods for bacterial detection, this study chose the same method used for viruses, that is, the multiplex real-time fluorescence PCR, for the detection of 16 pathogenic bacteria. In terms of age subgroups, bacterial infections were equally prevalent in the over 5-year age group, and there is also the possibility that older children had more exposure to different pathogens (Verbeke et al., 2019). However, there were no significant differences in other clinical features, laboratory indicators, or imaging outcome characteristics. The role of bacteria as pathogens of respiratory infections is debatable; our study samples were upper respiratory tract samples, and a positive bacterial test does not necessarily mean that it is the causative agent of the infection. It is necessary to combine factors, such as clinical manifestations and medical history characteristics, to determine whether the positive PCR result is caused by contamination, colonization, or pathogenic microorganisms (He et al., 2012). *Stenotrophomonas maltophilia* has been recognized in recent studies as an important nosocomial pathogen in immunocompromised and critically ill patients (De and Zhao, 2020; Nantachit et al., 2021; Zöllner et al., 2021) and as an important pathogen of hospital-acquired infections (Liu et al., 2018b; Wang et al., 2018). It is often isolated during invasive consultations (Liu et al., 2018a). In the present study, comparing the *S. maltophilia-*infected group with the uninfected group of HBoV-positive children, there were no significant differences in any of the indicators, except for some differences in Na levels. Since the sample collection in this study was at the time of admission, together with cross-examination of sample collection information and test results, the possibility of nosocomial bacterial infection or in-hospital contamination is ruled out. Although there is no clear conclusion regarding the pathogenic role of *S. maltophilia* infection from the analysis of clinical features and laboratory test results in this study, the detection rate of *S. maltophilia* (16.1%) in this study should be taken seriously. On this basis, we suggest strengthening the daily disinfection and sterilization of the hospital environment, raising the awareness of the standardized operation of invasive treatment in daily treatment, and cutting off the transmission pathway, to fundamentally eliminate the possibility of *S. maltophilia* causing harm to the hospitalized population.

The sequence and phylogenetic analyses indicated that 54 virus strains detected in this study belonged to HBoV1, the most predominant genotype in the global HBoV epidemic (Leitao et al., 2020; Netshikweta et al., 2020; Sharif et al., 2020). Although most of the viral strains clustered on close branches, there were still several strains in different branches with relatively longer genetic distances. In the present study, the other three variants (HBoV 2–4) were not identified, but it is possible that the remaining two HBoV-positive samples could be HBoV2–4. Currently, available research indicates that HBoV2–4 variants are commonly detected in fecal specimens of patients with gastroenteritis (Guido et al., 2016; Kenmoe et al., 2017; Madi and Al-Adwani, 2020), and the samples used in this study were all respiratory samples, which may explain the lack of HBoV 2–4 variants confirmed in this study. These findings are consistent with previous observations (Korsun et al., 2021). This finding also seems to confirm that HBoV1 is associated with respiratory tract infections. More than a decade has passed since the first discovery of HBoV, yet its role in respiratory infections remains unclear (Han et al., 2009; Koseki et al., 2012). We have tried to suggest the possibility that HBoV has little influence on the respiratory tract as a single virus, but affects some organs of the human body by combining the results of previous studies with the results of the present study (Gerna et al., 2007). This is an addition to the characteristics and less pathogenic nature of HBoV infection after prolonged shedding and remaining in the nasopharynx for several months (Guido et al., 2016; Korsun et al., 2021). In addition, it is well-known that a range of respiratory pathogens can inhibit viral replication by competing for resources, interfering with immune responses, or impacting viral proteins (Hobman et al., 2018; Dat et al., 2021). These phenomena suggest that HBoV requires coactivation with other pathogens that are not limited to common respiratory viruses, such as bacteria and fungi, to produce effects in the respiratory tract (Schildgen et al., 2008; Jartti et al., 2012). Additionally, research into the complex interactions between viruses and other pathogens can assist in understanding the epidemiology of respiratory pathogens, as well as planning infection control strategies to prevent their spread.

Overall, this study described a relatively comprehensive molecular etiology (viruses and bacteria), sequencing results, and patient clinical data. Although there was a lack of serological data on the studied population regarding viral infection, the different immunological levels of the studied population are yet to be categorized clearly. Our study demonstrates a useful retrospective application of clinically tested respiratory specimens, which should provide new insight into future exploration of current routinely collected coronavirus disease 2019 (COVID-19) clinical specimens. However, the limitations of this study include the following: First, our study subjects were hospitalized children with acute respiratory tract infections, which may lead to different results from those reported by children in the outpatient department at the same time. Second, this study used a relatively small sample size without severe cases and cannot represent a large population accurately, which may cause some errors in sampling. Finally, we used multiplex real-time fluorescence PCR for bacterial pathogen detection instead of the bacterial culture methods, which may reduce specificity to some extent while improving sensitivity.

### Future study

With the increasing use of multiplex real-time PCR in clinical laboratory settings, it is necessary to pay more attention to the detection rates of HBoV (types 1–4).

- A large number of clinical samples of HBoV single infection or mixed infection will be reserved for external cooperative research and development. With the improvement of the HBoV small intestinal epithelial cell infection model and infectious cloning technology in China, the demand for HBoV-positive specimens will be increasing.
- The actual prevalence of HBoV in Qingdao will be explored by further monitoring of HBoV infection in outpatients with fever, the general population (for example, the general population collected during COVID-19 screening as an asymptomatic infection group), and inpatients. This will help understand the broader data information on HBoV infection in the Qingdao population, and to set a more scientific survey time margin for the follow-up epidemiological tracing of specific groups, such as children with ARI and immunocompromised people. At the same time, this method is also applicable to other respiratory viruses.

## Data availability statement

The datasets presented in this study can be found in online repositories. The names of the repository/repositories and accession number(s) can be found in the article/Supplementary material.

## Ethics statement

The studies involving human participants were reviewed and approved by Regional Ethics Committee of the Qingdao Centers for Disease Control and Prevention Institutional Review Boards. Written informed consent to participate in this study was provided by the participants' legal guardian/next of kin.

## Author contributions

WW, ZL, and FZ conducted the overall design of the study. RG, RS, and SL conducted data collection and collation. WW, XS, ZS, and RL performed all the experiments. WW, KH, ZW, and XL wrote the manuscript. All authors contributed to the article and approved the submitted version.

## Funding

This work was funded by the Livelihood Science and Technology project of Qingdao (17-3-3-2-nsh) and science and technology benefit people demonstration and guidance project of Qingdao (20-3-4-51-nsh). The funders had no role in study design, data collection and analysis, the decision to publish, and in preparation of the manuscript.

## Conflict of interest

The authors declare that the research was conducted in the absence of any commercial or financial relationships that could be construed as a potential conflict of interest.

## Publisher's note

All claims expressed in this article are solely those of the authors and do not necessarily represent those of their affiliated organizations, or those of the publisher, the editors and the reviewers. Any product that may be evaluated in this article, or claim that may be made by its manufacturer, is not guaranteed or endorsed by the publisher.

## Abbreviations

ARIs: acute respiratory infections
HBoV: human bocavirus
*S. pneumoniae*: *Streptococcus pneumoniae*
*M. catarrhalis*: *Moraxella Catarrhalis*
*H. influenzae*: *Haemophilus influenzae*
*S. maltophilia*: *Stenotrophomonas maltophilia*
*S. aureus*: *Staphylococcus aureus*
*A. baumannii*: *Acinetobacter baumannii*
*M. tuberculosis*/*M. avium*: *Mycobacterium tuberculosis/Mycobacterium avium*
*M. pneumoniae*: *Mycoplasma pneumonia*
*K. pneumoniae*: *Klebsiella pneumoniae*
*B. pertussis*: *Bordetella pertussis*
*C. pneumonia*: *Chlamydia pneumonia*
*P. aeruginosa*: *Pseudomonas aeruginosa*
*L. pneumophila*: *Legionella pneumophila*
*S. marcescens*: *Serratia marcescens*
*B. parapertussis*: *Bordetella pertussis*
RSV: respiratory syncytial virus
HPIV: human parainfluenza virus
IFV: influenza virus
HAdV: human adenovirus
HRV: human rhinovirus
HEV: human enterovirus
HMPV: human metapneumovirus
HCoV: human coronavirus
LDH: lactate dehydrogenase
URE: urea
URIC: uric acid
CK-MB: creatine kinase isoenzyme
Mg: magnesium
WBC: white blood cells
RBC: red blood cell
ALT: glutamic-pyruvic transaminases
Na: sodium
AST: aspartate aminotransaminase
ADA: adenosine deaminase
EOS: eosinophil
Cys-C: cystatin-C.
