# Supplementary material for: Epidemiologic and clinical characteristics of human bocavirus infection in children hospitalized for acute respiratory tract infection in Qingdao, China
Source: Front Microbiol. 2022 Aug 10;13:935688. doi: 10.3389/fmicb.2022.935688 (PMC9399728; doi:10.3389/fmicb.2022.935688)
Supplement: Supplementary file 1 [file Data_Sheet_1.docx]

| **Supplementary Table 1.** The type of HBoV co-infected with other bacteria | | |
| --- | --- | --- |
|  | Type of coinfection | n (%) |
| one type of bacteria  (n=14,  25.0%) | *H. influenzae* | 3(5.4) |
|  | *A. baumannii* | 2(3.6) |
|  | *C. pneumonia* | 2(3.6) |
|  | *M. catarrhalis* | 2(3.6) |
|  | *S. maltophilia* | 2(3.6) |
|  | *S. pneumoniae* | 2(3.6) |
|  | *M. tuberculosis/M. avium* | 1(1.8) |
| two types of bacteria  (n=18,  32.1 %) | *S. pneumoniae+ M. catarrhalis* | 5(8.9) |
|  | S. pneumoniae+ *S. maltophilia* | 3(5.4) |
|  | *B. pertussis*+ *H. influenzae* | 2(3.6) |
|  | *S. pneumoniae*+*A. baumannii* | 2(3.6) |
|  | *C. pneumonia*+ *S. maltophilia* | 1(1.8) |
|  | *K. pneumoniae+ L. pneumophila* | 1(1.8) |
|  | *K. pneumoniae+ S. pneumoniae* | 1(1.8) |
|  | *M. catarrhalis + H. influenzae* | 1(1.8) |
|  | *S. aureus+ C. pneumonia* | 1(1.8) |
|  | *S. pneumoniae+ M. tuberculosis/M. avium* | 1(1.8) |
| three types of bacteria  (n=6,  10.7%) | *C. pneumonia+M. catarrhalis +M. tuberculosis/M. avium* | 1(1.8) |
|  | *H. influenzae+ A. baumannii+ S. marcescens* | 1(1.8) |
|  | *M. catarrhalis + H. influenzae+ S. pneumoniae* | 1(1.8) |
|  | *S. pneumoniae+ B. pertussis+ A. baumannii* | 1(1.8) |
|  | *S. pneumoniae+ M. catarrhalis + H. influenzae* | 1(1.8) |
|  | *S. pneumoniae+ S. aureus+ M. catarrhalis* | 1(1.8) |
| four types of bacteria  (n=1,  1.8%) | *K. pneumoniae+S. pneumoniae+ M. catarrhalis + M. tuberculosis/M. avium* | 1(1.8) |

| **Supplementary Table 2.** Comparison of the clinical characteristics of HBoV-positive patients between single infection and coinfections with high and low HBoV Ct-values | | | | | | |
| --- | --- | --- | --- | --- | --- | --- |
| Variable | High Ct-values+single infection(n=8) | High Ct-values+coinfections (n=29) | Low Ct-values+single infection(n=5) | Low Ct-values+coinfection(n=14) | χ²/Z/t | *P* |
| Gender |  |  |  |  |  | 0.547 |
| Male | 6(75.00%) | 13(44.83%) | 3(60.00%) | 7(50.00%) |  |  |
| Female | 2(25.00%) | 16(55.17%) | 2(40.00%) | 7(50.00%) |  |  |
| Age |  |  |  |  |  | 0.918 |
| <2 years old | 2(25.00%) | 12(41.38%) | 1(20.00%) | 4(28.57%) |  |  |
| 2-5 years old | 2(25.00%) | 8(27.59%) | 2(40.00%) | 4(28.57%) |  |  |
| ≥5 years old | 4(50.00%) | 9(31.03%) | 2(40.00%) | 6(42.86%) |  |  |
| Clinical features |  |  |  |  |  |  |
| Fever | 3(37.50%) | 10(34.48%) | 3(60.00%) | 7(50.00%) |  | 0.606 |
| Cough | 8(100.00%) | 28(96.55%) | 5(100.00%) | 14(100.00%) |  | 1.000 |
| Wheezing | 0(0.00%) | 7(24.14%) | 0(0.00%) | 5(35.71%) |  | 0.183 |
| Rales | 7(87.50%) | 19(65.52%) | 2(40.00%) | 11(78.57%) |  | 0.280 |
| Vomiting or diarrhea | 1(12.50%) | 1(3.45%) | 1(20.00%) | 2(14.29%) |  | 0.236 |
| WBC,10^9^/L | 8.25±2.24 | 8.41±3.26 | 7.78±2.94 | 9.11±4.41 | 0.238 | 0.869 |
| AST,U/L | 24.55(20.28,39.45) | 31.70(23.40,39.15) | 33.90(20.65,41.10) | 31.40(21.05,35.40) | 1.407 | 0.704 |
| ALT,U/L | 11.75(10.53,23.45) | 15.80(10.75,21.95) | 15.40(9.75,29.30) | 12.45(10.88,18.90) | 0.564 | 0.905 |
| CRP,mg/L | 8.29(0.83,27.84) | 2.57(0.62,12.16) | 2.77(0.50,47.25) | 4.82(0.50,13.28) | 0.880 | 0.830 |
| LDH,U/L | 277.50(261.50,286.75) | 279.00(249.00,371.50) | 316.00(208.50,471.50) | 275.00(225.50,322.25) | 0.672 | 0.880 |
| ADA,U/L | 19.00(18.00,24.25) | 20.00(17.00,24.00) | 20.00(17.00,27.00) | 20.50(18.00,23.00) | 0.595 | 0.898 |
| EOS,10^9^/L | 0.17(0.09,0.38) | 0.11(0.04,0.18) | 0.15(0.03,0.19) | 0.09(0.04,0.23) | 1.286 | 0.733 |
| RBC,10^9^/L | 4.56(4.25,4.86) | 4.37(4.13,4.65) | 4.43(4.22,5.30) | 4.34(4.11,4.70) | 0.695 | 0.559 |
| URE,mmol | 2.54(1.66,3.13) | 2.38(1.89,2.91) | 2.77(1.96,4.63) | 2.56(1.84,3.00) | 1.014 | 0.798 |
| URIC,umol/L | 252.00(234.50,284.75) | 218.00(184.50,249.50) | 175.00(157.50,266.00) | 235.00(166.00,260.50) | 1.059 | 0.374 |
| CK-MB,U/L | 28.95(21.20,35.68) | 25.20(19.70,37.50) | 26.70(20.35,61.80) | 26.20(17.00,36.43) | 0.191 | 0.979 |
| Cys-C,mg/L | 0.53(0.46,0.62) | 0.60(0.51,0.83) | 0.64(0.51,0.68) | 0.60(0.52,0.84) | 2.900 | 0.407 |
| HCO_3_,mmol/L | 22.95(21.58,23.58) | 20.00(18.00,22.00) | 22.50(18.65,23.00) | 21.05(16.75,23.35) | 7.111 | 0.068 |
| Na,mmol/L | 139.10(138.48,140.90) | 139.00(137.45,140.50) | 139.90(138.90,141.95) | 138.55(137.90,141.38) | 0.695 | 0.559 |
| Mg,mmol/L | 0.97(0.92,1.01) | 1.04(0.96,1.14) | 1.06(0.99,1.21) | 1.10(1.03,1.20) | 10.069 | 0.018* |
| Hospitalization days | 7.50(5.00,10.50) | 8.00(6.00,8.50) | 12.00(6.00,13.50) | 7.00(6.00,9.00) | 2.292 | 0.514 |
| Increased lung markings | 4(50.00%) | 20(68.97%) | 3(60.00%) | 3(21.43%) |  | 0.028* |
| Patchy opacities | 4(50.00%) | 15(51.72%) | 3(60.00%) | 10(71.43%) |  | 0.635 |
| Linear opacities | 3(37.50%) | 1(3.45%) | 1(20.00%) | 0(0.00%) |  | 0.019* |
| Air bronchogram sign | 1(12.50%) | 2(6.90%) | 1(20.00%) | 1(7.14%) |  | 0.574 |
| *P values ≤ 0.05 were considered to be statistical significant.  Fever: T ≥ 37.5℃ (axillary temperature)  Reference levels: WBC(4~10), AST(15~40), ALT(9-50), CRP(0~5), LDH(120~250), ADA(4~18), EOS(0.02~0.52), RBC(3.75~5.5), URE(3.1~8.0), URIC(89.2~416), CK-MB(0~17), Cys-C(0.6~1.55), HCO_3_(23~31), Na(137~147), Mg(0.75~1.02) | | | | | | |

| **Supplementary Table 3.** Clinical characteristics of HBoV-positive patients infected alone or in combination with other viruses | | | | |
| --- | --- | --- | --- | --- |
| Variable | Single-infection (n=42) | Mixed-infection(n=14) | χ²/Z/t | *P* |
| Gender |  |  | 4.029 | 0.045* |
| Male | 25(59.52%) | 4(28.57%) |  |  |
| Female | 17(40.48%) | 10(71.43%) |  |  |
| Age |  |  |  | 0.686 |
| <2 years old | 14(33.33%) | 5(35.71%) |  |  |
| 2-5 years old | 11(26.19%) | 5(35.71%) |  |  |
| ≥5 years old | 17(40.48%) | 4(28.58%) |  |  |
| Clinical features |  |  |  |  |
| Fever | 17(40.48%) | 6(42.86%) |  | 1.000 |
| Cough | 41(97.62%) | 14(100.00%) |  | 1.000 |
| Wheezing | 6(14.29%) | 6(42.86%) | 3.535 | 0.060 |
| Rales | 29(69.05%) | 10(71.43%) | 0.000 | 1.000 |
| Vomiting or diarrhea | 5(11.90%) | 0(0.00%) |  | 0.316 |
| WBC,10^9^/L | 8.97±3.37 | 7.12±3.13 | 1.811 | 0.076 |
| AST,U/L | 26.70(21.70,35.85) | 36.05(31.35,40.90) | 2.195 | 0.028* |
| ALT,U/L | 13.75(10.63,21.93) | 14.80(10.70,28.03) | 0.397 | 0.691 |
| CRP,mg/L | 3.79(0.50,18.88) | 2.47(0.59,7.39) | 0.791 | 0.429 |
| LDH,U/L | 277.50(247.00,317.00) | 291.00(247.25,376.50) | 0.568 | 0.570 |
| ADA,U/L | 19.00(17.50,22.25) | 22.50(20.00,26.25) | 2.593 | 0.010* |
| EOS,10^9^/L | 0.16(0.07,0.24) | 0.04(0.01,0.11) | 2.558 | 0.011* |
| RBC,10^9^/L | 4.54±0.48 | 4.20±0.43 | 2.489 | 0.016* |
| URE,mmol | 2.70(1.97,3.10) | 2.06(1.68,2.75) | 1.448 | 0.148 |
| UA,umol/L | 226.36±50.77 | 209.64±63.57 | 1.00 | 0.322 |
| CK-MB,U/L | 25.10(19.30,34.63) | 28.55(21.50,38.50) | 0.927 | 0.354 |
| Cys-C,mg/L | 0.58(0.48,0.66) | 0.82(0.69,0.94) | 3.124 | 0.002* |
| HCO_3_,mmol/L | 21.65(19.93,22.93) | 18.40(15.65,20.45) | 2.744 | 0.006* |
| Na,mmol/L | 139.13±2.18 | 139.41±1.55 | 0.457 | 0.650 |
| Mg,mmol/L | 1.04(0.97,1.13) | 1.04(0.95,1.12) | 0.616 | 0.538 |
| Hospitalization days | 7.50(6.00,9.25) | 7.00(6.00,8.00) | 1.13 | 0.258 |
| Imaging results confirmed pneumonia | 25(59.52%) | 3(21.43%) | 6.095 | 0.014* |
| Increased lung markings | 21(50.00%) | 9(64.29%) | 0.862 | 0.353 |
| Patchy opacities | 27(64.29%) | 5(35.71%) | 3.500 | 0.061 |
| Linear opacities | 5(11.90%) | 0(0.00%) |  | 0.316 |
| Air bronchogram sign | 3(7.14%) | 2(14.29%) |  | 0.590 |
| *P values ≤ 0.05 were considered to be statistical significant.  Fever: T ≥ 37.5℃ (axillary temperature)  Reference levels: WBC(4~10), AST(15~40), ALT(9-50), CRP(0~5), LDH(120~250), ADA(4~18), EOS(0.02~0.52), RBC(3.75~5.5), URE(3.1~8.0), URIC(89.2~416), CK-MB(0~17), Cys-C(0.6~1.55), HCO_3_(23~31), Na(137~147), Mg(0.75~1.02) | | | | |

| **Supplementary Table 4.** Clinical characteristics of HBoV-positive patients with or without bacteria | | | | |
| --- | --- | --- | --- | --- |
| Variable | With bacteria (n=39) | Without bacteria (n=17) | χ²/Z/t | *P* |
| Gender |  |  |  |  |
| Male | 18(46.15%) | 11(64.71%) | 1.632 | 0.201 |
| Female | 21(53.85%) | 6(35.29%) |  |  |
| Age |  |  |  |  |
| <1 years old | 8(20.51%) | 3(17.65%) | 4.902 | 0.297 |
| 1-2 years old | 7(17.95%) | 1(5.88%) |  |  |
| 2-3 years old | 6(15.38%) | 1(5.88%) |  |  |
| 3-4 years old | 4(10.26%) | 5(29.41%) |  |  |
| ≥5 years old | 14(35.90%) | 7(41.18%) |  |  |
| Clinical features |  |  |  |  |
| Fever | 15(38.46%) | 8(47.06%) | 0.362 | 0.548 |
| Cough | 38(97.44%) | 17(100.00%) | 0.444 | 0.505 |
| Wheezing | 11(28.21%) | 1(5.88%) | 2.304 | 0.129 |
| Rales | 27(69.23%) | 12(70.59%) | 2.848 | 0.091 |
| Vomiting or diarrhea | 3(7.69%) | 2(11.76%) | 0.000 | 1.000 |
| WBC,10^9^/L | 8.73±3.74 | 7.99±2.40 | 0.881 | 0.383 |
| AST,U/L | 31.40(22.90,38.20) | 33.20(21.15,40.05) | 0.196 | 0.845 |
| ALT,U/L | 15.80(11.10,22.00) | 11.80(10.55,20.90) | 0.775 | 0.438 |
| CRP,mg/L | 2.57(0.50,10.40) | 5.74(0.55,26.22) | 1.149 | 0.250 |
| LDH,U/L | 279.00(244.00,369.00) | 281.00(256.50,312.50) | 0.089 | 0.929 |
| ADA,U/L | 20.00(18.00,23.00) | 19.00(18.00,24.50) | 0.107 | 0.915 |
| EOS,10^9^/L | 0.10(0.05,0.22) | 0.15(0.05,0.21) | 0.339 | 0.735 |
| RBC,10^9^/L | 4.42±0.48 | 4.52±0.42 | 0.732 | 0.467 |
| URE,mmol | 2.55±0.91 | 2.69±1.18 | 0.492 | 0.625 |
| UA,umol/L | 220.33±45.85 | 226.41±71.08 | 0.324 | 0.749 |
| CK-MB,U/L | 25.20(19.40,37.00) | 28.60(21.80,36.00) | 0.758 | 0.449 |
| Cys-C,mg/L | 0.58(0.53,0.78) | 0.59(0.48,0.72) | 0.508 | 0.611 |
| HCO_3_,mmol/L | 20.90(18.20,22.10) | 22.70(18.60,23.35) | 1.631 | 0.103 |
| Na,mmol/L | 139.89±2.17 | 139.90±1.49 | 1.739 | 0.088 |
| Mg,mmol/L | 1.07(1.00,1.14) | 1.02(0.96,1.06) | 1.703 | 0.088 |
| Hospitalization days | 7.00(6.00,9.00) | 8.00(6.00,11.00) | 0.794 | 0.427 |
| Imaging results confirmed pneumonia | 19(48.72%) | 9(52.94%) | 0.084 | 0.771 |
| Increased lung markings | 19(48.72%) | 8(47.06%) | 0.013 | 0.909 |
| Patchy opacities | 17(43.59%) | 9(52.94%) | 0.386 | 0.535 |
| Linear opacities | 1(2.6%) | 4(23.53%) | 2.105 | 0.147 |
| Air bronchogram sign | 1(2.56%) | 3(17.65%) |  | 0.546 |
| *P values ≤ 0.05 were considered to be statistical significant.  Fever: T ≥ 37.5℃ (axillary temperature)  Reference levels: WBC(4~10), AST(15~40), ALT(9-50), CRP(0~5), LDH(120~250), ADA(4~18), EOS(0.02~0.52), RBC(3.75~5.5), URE(3.1~8.0), URIC(89.2~416), CK-MB(0~17), Cys-C(0.6~1.55), HCO_3_(23~31), Na(137~147), Mg(0.75~1.02) | | | | |

| **Supplementary Table 5.** Clinical characteristics of HBoV-positive patients with or without *S.maltophilia* | | | | |
| --- | --- | --- | --- | --- |
| Variable | With *S. maltophilia* (n=7) | Without *S. maltophilia* (n=49) | χ²/Z/t | *P* |
| Gender |  |  |  |  |
| Male | 4(57.17%) | 25(51.02%) | 0.000 | 1.000 |
| Female | 3(42.86%) | 24(48.98%) |  |  |
| Age |  |  |  |  |
| <2 years old | 2(28.57%) | 24(48.98%) |  |  |
| 2-5 years old | 1(14.29%) | 9(18.37%) |  |  |
| ≥5 years old | 4(57.14%) | 16(32.65%) |  |  |
| Clinical features |  |  |  |  |
| Fever | 4(57.14%) | 19(38.78%) |  | 0.429 |
| Cough | 7(10.00%) | 48(97.96%) |  | 1.000 |
| Wheezing | 2(28.57%) | 10(20.41%) | 0.000 | 1.000 |
| Rales | 2(28.57%) | 37(75.51%) |  | 0.022* |
| Vomiting or diarrhoea | 1(14.29%) | 4(8.16%) |  | 0.501 |
| WBC,10^9^/L | 6.96±3.82 | 8.72±3.30 | 1.297 | 0.200 |
| AST,U/L | 34.60(31.40,38.80) | 30.20(22.20,38.25) | 1.041 | 0.298 |
| ALT,U/L | 15.80(12.50,27.70) | 13.70(10.30,21.95) | 1.078 | 0.281 |
| CRP,mg/L | 2.42(0.65,3.89) | 3.69(0.50,18.18) | 0.599 | 0.549 |
| LDH,U/L | 279.00(255.00,372.00) | 280.00(240.00,324.50) | 0.520 | 0.603 |
| ADA,U/L | 22.00(20.00,26.00) | 19.00(18.00,23.00) | 1.754 | 0.079 |
| EOS,10^9^/L | 0.08(0.01,0.05) | 0.12(0.05,0.19) | 0.074 | 0.941 |
| RBC,10^9^/L | 4.35±0.77 | 4.47±0.41 | 0.376 | 0.719 |
| URE,mmol | 2.72(2.07,3.34) | 2.50(1.82,2.96) | 0.954 | 0.340 |
| UA,umol/L | 215.43±49.20 | 223.14±55.20 | 0.350 | 0.728 |
| CK-MB,U/L | 23.00(20.00,42.90) | 26.70(19.70,36.00) | 0.186 | 0.853 |
| Cys-C,mg/L | 0.57(0.48,0.88) | 0.59(0.50,0.76) | 0.248 | 0.804 |
| HCO_3_,mmol/L | 18.50(16.80,22.) | 21.60(18.75,22.85) | 1.363 | 0.173 |
| Na,mmol/L | 137.90±0.80 | 139.38±2.09 | 3.484 | 0.002* |
| Mg,mmol/L | 1.07(1.02,1.18) | 1.04(0.96,1.11) | 1.079 | 0.281 |
| Hospitalization days | 7.00(5.00,9.00) | 7.00(6.00,9.00) | 0.314 | 0754 |
| Imaging results confirmed pneumonia | 2(28.6%) | 26(53.1%) | 0.653 | 0.419 |
| Increased lung markings | 4(57.14%) | 26(53.06%) |  | 1.000 |
| Patchy opacities | 2(28.57%) | 17(34.69%) |  | 1.000 |
| Linear opacities | 3(42.86%) | 29(59.18%) |  | 0.447 |
| Air bronchogram sign | 1(14.29%) | 4(8.16%) |  | 0.501 |
| *P values ≤ 0.05 were considered to be statistical significant.  Fever: T ≥ 37.5℃ (axillary temperature)  Reference levels: WBC(4~10), AST(15~40), ALT(9-50), CRP(0~5), LDH(120~250), ADA(4~18), EOS(0.02~0.52), RBC(3.75~5.5), URE(3.1~8.0), URIC(89.2~416), CK-MB(0~17), Cys-C(0.6~1.55), HCO_3_(23~31), Na(137~147), Mg(0.75~1.02) | | | | |
